# Sleep and Sound Sleepers

**Published:** 1885-04

**Authors:** 


### SLEEP AND SOUND SLEEPERS.

A Case of supposed suicide which recently occurred in a quiet Connecticut village caused intense local interest. The person concerned was the victim of a curious disease—we know of no better name for it—which caused him to sleep for months at a time. When he awoke he was much depressed to know that he was the subject of such a strange malady, and it is thought that this led him to commit the rash act.

Sleep is nearly as great a puzzle as it ever was. Much has been learned concerning the bodily peculiarities manifested during this portion of our existence; but all whose opinions are best worth listening to, frankly admit that they are only on the threshold of the subject yet. Why, for instance, can some men maintain their bodily and mental vigor with so small an amount of sleep as falls to their share? Lord Brougham and many other great statesmen and persons of note are known to have been content with a marvelously small amount of sleep. Frederick the Great is said to have allowed himself ouly five hours; John Hunter, five hours; General Elliott, the hero of Gibraltar, four hours; while Wellington, during the Peninsular War, had still less.

On the other hand, how are we to account for the cormorant sleepers? Dr. Moivre, the mathematician, could—though it is to be hoped he did not—sleep twenty hours out of the twenty-four. Quinn, the actor, sometimes slept twenty-four hours at a stretch, Dr. Reid, the mataphysician, could so manage that one potent meal, followed by one long and sound sleep, would last him for two days. In the middle of the last century a young Frenchwoman at Toulouse had for six months or more, fits of lengthened sleep varying from three to fifteen days each. About the same time a girl at Newcastle-on-Tyne slept fourteen weeks without waking, and the waking procees occupied three days to complete it. Dr. Blanchet, of Paris, mentions the case of a lady who slept for twenty days together when she was about eighteen years of age, fifty when she was twenty, and later had nearly a whole year's sleep. from Easter Sunday, 1862, to March, 1863; during this long sleep, which physicians called hysterical coma, she was fed with milk and soup, one of her front teeth being extracted to obtain entrance to her mouth. Another very notable instance was that of Samuel Chilton Timsbury. recorded in the Transactions of the Royal Society. In the year 1694 he slept for a month and no one could wake him. Later in the same year he had a four months' sleep, from April 9th to August 7th ; he awoke, dressed, and went out into the fields,—where he worked as a laborer—and found his compauions reaping the wheat which he had helped to sow the day before his long nap ; it was not until then that he knew of his sleep having exceeded the usual duration of a few hours. He went to sleep again on the 17th and did not awake until November 19th, notwithstanding the vigorous application of hellebore and sal ammoniac to his nostrils, and bleeding to the extent of fourteen ounces. He woke, asked for bread and cheese, but went off to sleep again before it could be brought to him, taking another snooze which lasted until the end of January. It is not recorded that he had any more of these strange relapses after that. The mere contemplation of such cases is enough to make one sleepy.

There are instances of sleep so intensely deep as to deprive the sleeper of all sense of pain. The records of the Infirmary in Bristol, England, furnish a striking illustration of this. One cold night a tramp lay down near a lime-kiln and went to sleep. One foot must have been near the fire hole close to the kiln, for during the night the foot and ankle were so completely burned away as to leave nothing but black cinder and calcined ash. He did not wake till the kiln man roused him next morning, nor did he know what had occurred until he looked down at his charred stump. He denied that he had taken any drug or liquor, and there was no evidence whatever that he was under the influence of either. He died some weeks later of gangrene.- *Mass. Med. Journal*

